# Manipulation Tasks in Hazardous Environments Using a Teleoperated Robot: A Case Study at CERN

**DOI:** 10.3390/s23041979

**Published:** 2023-02-10

**Authors:** Cosimo Gentile, Giacomo Lunghi, Luca Rosario Buonocore, Francesca Cordella, Mario Di Castro, Alessandro Masi, Loredana Zollo

**Affiliations:** 1 Centro Protesi Inail, Vigorso di Budrio, 40054 Bologna, Italy; 2 Unit of Advanced Robotics and Human-Centred Technologies, Università Campus Bio-Medico di Roma, 00128 Rome, Italy; 3 Survey, Measurement and Mechatronics (EN-SMM) Group, CERN, 1217 Geneva, Switzerland

**Keywords:** mobile robots, teleoperation, force control, slippage detection, industrial gripper

## Abstract

Remote robotic systems are employed in the CERN accelerator complex to perform different tasks, such as the safe handling of cables and their connectors. Without dedicated control, these kinds of actions are difficult and require the operators’ intervention, which is subjected to dangerous external agents. In this paper, two novel modules of the CERNTAURO framework are presented to provide a safe and usable solution for managing optical fibres and their connectors. The first module is used to detect touch and slippage, while the second one is used to regulate the grasping force and contrast slippage. The force reference was obtained with a combination of object recognition and a look-up table method. The proposed strategy was validated with tests in the CERN laboratory, and the preliminary experimental results demonstrated statistically significant increases in time-based efficiency and in the overall relative efficiency of the tasks.

## 1. Introduction

CERN [1,2,3], European Center for Nuclear Research, located in Geneva, Switzerland, hosts more than 50 km of underground particle accelerators [4], including the Large Hadron Collider (LHC), the biggest particle accelerator in the world (it is approximately 27 km in circumference [5]), which contains a huge variety of scientific equipment. The size of the LHC poses continuous challenges concerning both its design and construction, and its maintenance, a critical point to ensure the regular operation of the experimental facility. Nevertheless, CERN experimental areas located underground present hazardous characteristics: the presence of radiation, high magnetic fields and possible lack of oxygen. Therefore, ensuring safe personnel access to the accelerator facilities can be challenging. The use of robotic systems in hazardous environments allows personnel safety to be ensured. Moreover, more accurate and detailed data about the environment can be collected during robotic operation, resulting in more opportunities for more accurate and precise operation [6]. Teleoperated robotic platforms can perform some of the maintenance tasks more safely and reliably than humans. The maintenance of the LHC includes a wide list of different tasks: visual inspection, screwing, welding, disassembling, reassembling and many others. For each of these tasks, different robots are needed, with sensors and tools, to face every situation.

Performing a stable grasp is the main challenge for most robotic manipulators, due to the need to avoid the application of excessive gripping force and prevent slippage as well as any possible damage to the object. The variability of the properties of the objects involved in activities of daily living makes grasping difficult when the robot handles novel objects without having prior information. Too much force could deform or damage the object or the gripper fingers, while too little pressure could let it slip or drop during displacements.

By using a robot gripper, dexterous handling can be achieved using information measured with sensors, such as force sensors, torque sensors, slip sensors, contact sensors, etc. [7]. The incipient slip information in human grasping [8] allows one to quickly adjust the gripping force in response to the object characteristics perceived upon initial contact [8,9]. In [10], an adaptive closed-loop grasping algorithm for novel objects is implemented on a robotic gripper instrumented with a force-sensing resistor (FSR) sensor and a laser-based slip sensor. This algorithm can immobilize a novel object within the fingers of the gripper with minimal deformation by estimating the exact minimal grasping force. In [11], a slipping avoidance algorithm is proposed to allow the robot to react to both linear and rotational slippage of the grasped object. The object grasped with a one-DoF gripper, provided by a six-axis force/tactile sensor, can be safely lifted with given orientation and information about its centre of gravity, and uncertain friction properties are not necessary. In [12], a model-free intelligent fuzzy sliding mode control strategy is employed in an ad hoc developed gripper sensorised with an FSR on the fingers. Slip information is obtained after three consecutive force variations exceeding a specific threshold. The gripper can dexterously pick and place various objects by using stiffness information and generating an appropriate grasping force, and the anti-slip control strategy can adjust the grasping force online to avoid object slippage. In [13], two different algorithms for controlling the grasping force are implemented in a one-DoF parallel gripper sensorised with six-axis force/tactile sensors. The first algorithm is aimed at avoiding both linear and rotational slippage [14] by using, on the grasped object, as little force as possible. The controller does not need information about the properties of the object to be grasped. The second algorithm achieves the pivoting task, i.e., a controlled rotation of the grasped object to change its orientation without releasing the object. In [15], gecko-inspired adhesives are mounted on the tips of commercial gripper fingers to delicately grasp objects by using little force. The gripper is equipped with tactile sensors [16]. Information about external forces and moments derive from a sensor on the robot’s wrist. The gecko adhesive characteristic is increased adhesion in proportion to the applied shear load. The knowledge of gripper performance combined with the gecko-inspired adhesive properties significantly reduces the overall demand for gripper actuators; then, smaller types can be used to lighten tools on robotic arms. In [17], the Vibratory Finger Manipulator, a simple and affordable mechanism based on the stick–slip phenomenon, where the application of vibrations enables friction to be actively controlled, is proposed to potentially augment the capabilities of any generic parallel jaw gripper. This approach generates propagation force onto the touched object, allowing the manipulation of the object to be achieved with accuracy of less than 2 mm.

During the grasping and lifting of various objects, visual cues and beforehand-gained knowledge allow humans to prepare for the imminent grasp by adapting the fingertip force based on the real object weight [18]. Humans evaluate the object weight to grasp using vision as an initial estimation; successively, they use tactile afferent control to improve the grasping precision [19]. Different approaches have been developed to estimate the object’s weight. In [20], active thermography and custom multi-channel neural networks are used to classify the density property. The approach is capable to estimate the weight of the unknown object by evaluating its volume. In [21], the estimation of the object weight is obtained by measuring the currents flowing in the gripper motor servos and by using linear regression between these and the weight values; the results show an estimation of the weight of the object with an average error of 22.42%.

For the execution of a task (e.g., plugging in/unplugging a connector), information on the object weight is not sufficient. Different solutions have been studied to overcome this problem. In [22], a gripper is proposed for the accurate alignment and holding of the position between the cable and the gripper. The method for plugging in the connector needs information on the exact connector position of the gripper. If the tolerance between the connectors is larger than the error position of the robot arm, the connection can be achieved using the position control of conventional industrial robots, without using force information. In [23], different solutions are presented to increase the degree of autonomy of robots involved in underwater intervention missions. In particular, an explored task involves the plugging in/unplugging of the hot stab connector. The whole procedure allows one to achieve the completion of the task, but no information is reported about the control of the grasping force. Just two parts of the presented algorithm to perform the task are notable: “Close the gripper completely; Reach the plug waypoint [...] This waypoint is 1 cm deeper than the manipulation waypoint because sometimes it is necessary to exert a bit of force to be sure that the connector has entered completely. The execution of known and pre-imposed actions is allowed by a controlled environment; again, a new unexpected condition could be unmanaged”.

In the case of the handle of optical fibres and their connectors, the interventions at CERN cannot be performed appropriately without a force control law that regulates the force applied by the grippers. The object could fall or the task could not be performed, and human intervention in hazardous environments would be necessary.

**Figure 1:**
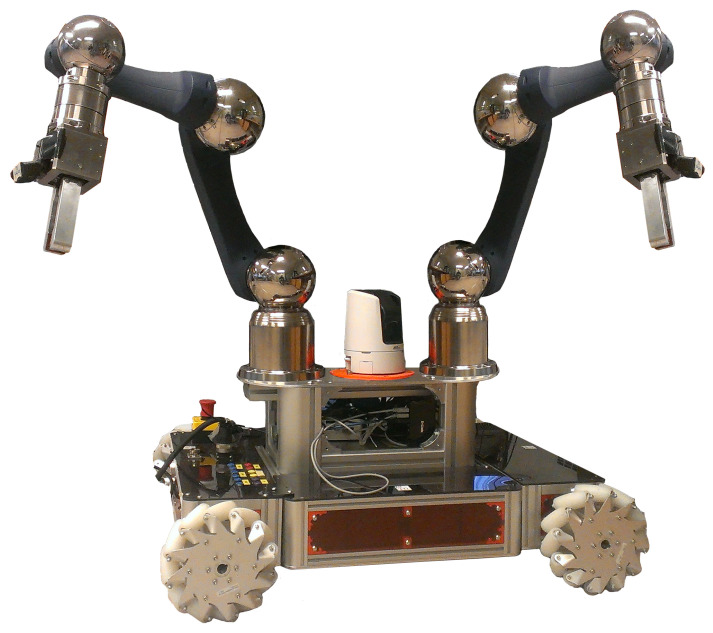
Dual-arm CERNBot.

The aim of this work was to overcome this limitation by providing novel modules of the CERNTAURO [24] framework already present at CERN and used for other types of interventions: (i) the first module is a touch-and-slippage algorithm [25], and (ii) the second one, force-and-slippage control [26] developed for prosthetic hands and adapted for this work. CERNBot [27], a robotic platform (Figure 1) built at CERN, was used in the dual-arm configuration. On each arm, an industrial gripper equipped with two ad hoc developed fingers was munted.

The strategy also includes an object recognition module (ORM) to recognize metallic objects (e.g., connectors, sockets and patch panels) [28] and choose a force reference from a table for the gripper to perform the desired task. The tasks were performed in a teleoperated way to recreate the conditions of a real intervention in hazardous environments. The combination of the two novel modules and the ORM was employed to safely perform the plugging in and the unplugging of an optical fibre connector by applying the correct force to the connector for its management and to prevent any slipping during the execution of the tasks.

## 2. Materials and Methods

### 2.1. Touch-and-Slippage Detection Algorithm

The algorithm is able to detect (i) the first touch between the sensor and an object and (ii) slippage events. For removing the electronic noise from setup components, the mean value, $v_{mean}$, of the FSR conditioned signal (i.e., force or voltage), $v_{i}$, was computed for every 5 samples.

(1) $$v_{mean}=\frac{1}{5}\sum_{i=1}^{5}v_{i}$$

The steps of the touch identification procedure are the following:

(1) Computation of the average value on 10 samples of the voltage signal in the resting (calibration) period. In the calibration period, no force variation is detected, and $v_{rest}$ represents the mean of background noise magnitude.
(2) Comparison of $v_{rest}$ with $v_{mean}$ to obtain a mean voltage error.

(2) $$\Delta v=v_{rest}-v_{mean}$$

Contact with the object is detected if Δv is greater than the minimum voltage variation, δ, measured by the sensor when pressed.

(3) Δv>δ,touch=1;

Next, the value of $v_{mean}^{\prime }$, i.e., the voltage signal positive derivative, is computed. Only the positive derivative is considered, since the negative value corresponds to a pressure increment on the FSR.

(4) $$v_{mean}^{\prime }=\frac{d}{dt}v_{mean}$$

The derivative of the mean values between two consecutive 5-sample sets is computed and compared with a threshold α established using the ROC curve [29], ensuring the discrimination between true and false-positive slippage events. A binary value (called slip) is set to 1 when $v_{mean}^{\prime }$ is higher than the threshold (i.e., the slippage occurs); this is 0 otherwise.

(5) $$\left\{\begin{array}{c} v_{mean}^{\prime }\geq \alpha ,slip=1\\ v_{mean}^{\prime }<\alpha ,slip=0 \end{array}\right.$$

The proposed approach can be applied both on voltage and force signals by simply choosing the negative or positive derivative of the signal. In this work, 100 Hz was the sampling rate for calculating touch and slippage.

### 2.2. Force Reference Estimation

The normal force reference, $F_{d}$, is determined by analysing the gripper grasping and manipulating a set of objects (i.e., plugging in and unplugging connectors) available in hazardous environments. For each connector mounted on test benches in the laboratory, the gripper of the robot repeated the grasping and the operation of plugging in/unplugging three times. The grasping forces were acquired with the FSR sensors embedded in the fingers, and the mean values are reported in Table 1.

Then, the camera mounted on the robot (used to perform interventions in a teleoperated manner) is used to recognize the object to grasp and to select the corresponding reference normal force, $F_{d}$, from a table.

The strategy also includes the ORM [28], a module based on a deep learning-based module for object recognition [30] that allows metallic objects to be recognised (e.g., connectors, sockets and patch panels) according to non-textured attributes. The module is based on region-based convolutional neural networks (Faster-RCNN) already pre-trained in COCO [31]. The neuronal model chosen for this work to detect a large number of metallic parts was ResNet-101 [32].

### 2.3. Force-and-Slippage Control

**Figure 2:**
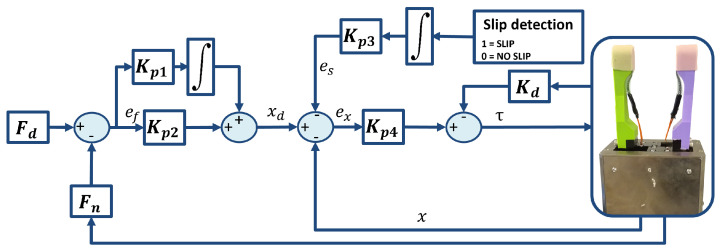
Force-and-slippage control law.

For this work, force-and-slippage control used in the prosthetic field [26] with a touch-and-slippage detection algorithm [25] developed by the authors was chosen (Figure 2). If the proposed strategy works well in a challenging area such as prosthetics, where stability and reduced response times are required, good functioning is also expected in other less stringent but equally challenging areas. The coordination of the fingers was not used, because the gripper only had one motor for moving the two fingers.

$F_{n}$, i.e., the sum of the components of the normal forces applied by the gripper fingertips [33] on the object surface and acquired with force sensors, is subtracted from the reference force, $F_{d}$, to obtain a force error, $e_{f}$, that has to be minimized by the control. Then, reference position $x_{d}$ is obtained with proportional–integrative (PI) force control,

(6) $$x_{d}=K_{p1}\int_{0}^{t_{f}}\left(F_{d}-F_{n}\right)dt+K_{p2}\left(F_{d}-F_{n}\right)$$

and is compared with the actual position, *x*. $K_{p1}$ and $K_{p2}$ are the controller gains, and $t_{f}$ is the final integration time, while $K_{p1}=3$ and $K_{p2}=2.7$ are the controller gains obtained with a trial-and-error procedure, assuming them to be much higher than the stiffness of the sensorised fingers. An additional contribution, $e_{s}$, is considered to manage slippage events [34,35]. Therefore, the position error is

(7) $$e_{x}=x_{d}-x-e_{s}$$

where

(8) $$es=K_{p3}\int_{0}^{t_{f}}slip,$$

slip is the binary signal equal to 0 obtained using the touch-and-slippage detection algorithm (Section 2.1) and $K_{p3}=1.34$ is a constant regulating the $e_{s}$ weight in the control obtained with a trial-and-error procedure. The integration of this signal guarantees an increment in the applied grasping force in the presence of slippage [34].

The so-obtained position error $e_{x}$ should be reduced to zero by PD control written as

(9) $$\tau =K_{p4}\left(x_{d}-x-e_{s}\right)-K_{D}\dot{x}$$

where τ is the torque; *x*, $\dot{x}$ and $\ddot{x}$ are position, velocity and acceleration derived from the motor sensors; $K_{p4}=2.7$ and $K_{D}=0.42$ are the proportional and the derivative gains, respectively.

### 2.4. CERNTAURO Framework

**Figure 3:**
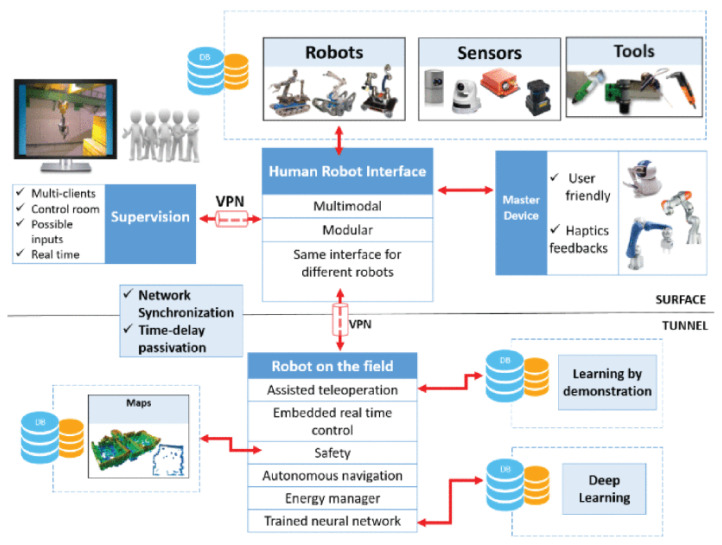
CERNTAURO framework.

The project in [24] (Figure 3) aims at creating a modular framework adaptable to specific intervention needs, to be upgraded accordingly as new features need to be integrated. The CERNTAURO framework is adaptable to different robots thanks to a configuration layer that takes into account several factors, such as type of hardware, communication layer and user needs, and can perform unmanned tasks in hazardous and semi-structured environments.

### 2.5. CERNBot

CERNBot (Figure 1) is a modular and flexible robotic platform built at CERN for complex interventions in the presence of hazards, such as ionization radiation. It is made of two robotic arms (Schunk) installed on a mobile platform. CERNBot uses standard industry components for most of its electronic and control hardware, making it a constantly evolving platform, as hardware is upgraded by the manufacturer. Further details can be found in [27]. For this application, an industrial gripper (already used and chosen among the available ones) was selected and installed on the robotic arm.

### 2.6. Multimodal Human–Robot Interface

The human–robot interface (HRI) presented in [36] has been developed for remote robotic intervention in hazardous environments, and it is part of the CERNTAURO framework. According to its definition, a multimodal interface provides different modalities for user interaction: the interaction domain of user and interface, and the interaction domain of user and robot.

In this work, the interaction between the user and the interface and that between the user and the robot were performed with the keyboard.

### 2.7. Experimental Setup and Protocol

**Figure 4:**
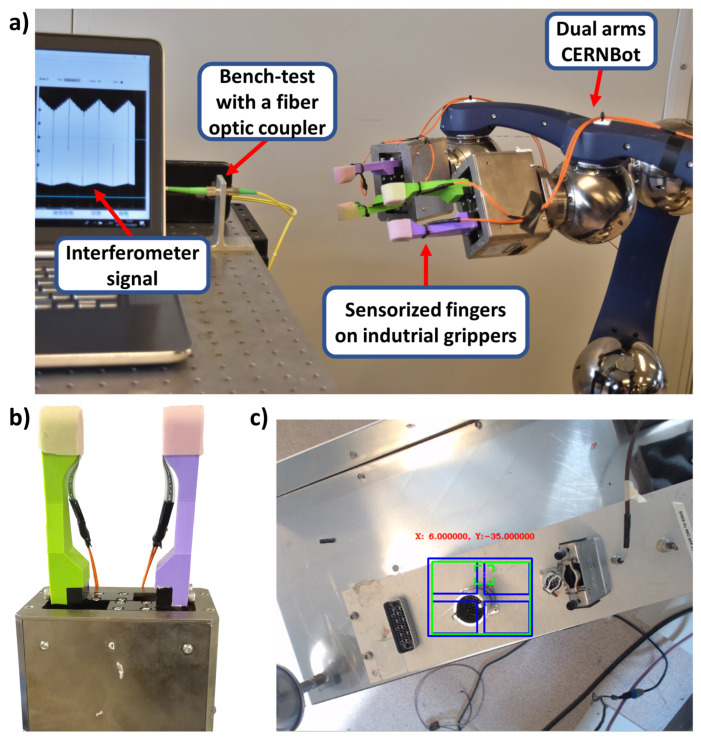
(**a**) Setup for experimental validation. (**b**) Particular of the gripper with the two ad hoc fingers. (**c**) Object recognition for the force reference of choice.

CERNBot in the dual-arm configuration (right side of the Figure 4a) was positioned in front of a table where a test bench with a fibre optic coupler (in the centre of the Figure 4a) was mounted.

Two commercial industrial grippers (Universal gripper PG; size, 70; Schunk, Lauffen am Neckar, Germany) [33], with fingers realized with PLA, instrumented with two FSR sensors Model 402 (Figure 4 [37]) and covered with silicon to increase the grip, were mounted on the robotic arms of CERNBot. This kind of gripper has one motor to drive the ball screw via a toothed belt drive; then, the rotational movement is transformed into a linear movement by base jaws mounted on the spindle nuts. The position was measured using an embedded encoder sensor. The position was the same for both fingers due to the parallel mechanisms [33].

The user was sitting in front of a monitor and was asked to perform the teleoperated tasks by following the whole operation using the camera mounted on the robot. Communication between the user and the robot was performed by means of an ethernet cable. The user manually chose the type of connector to recognize and handle, and the correct force value was automatically chosen from the table and set in the control.

**Figure 5:**
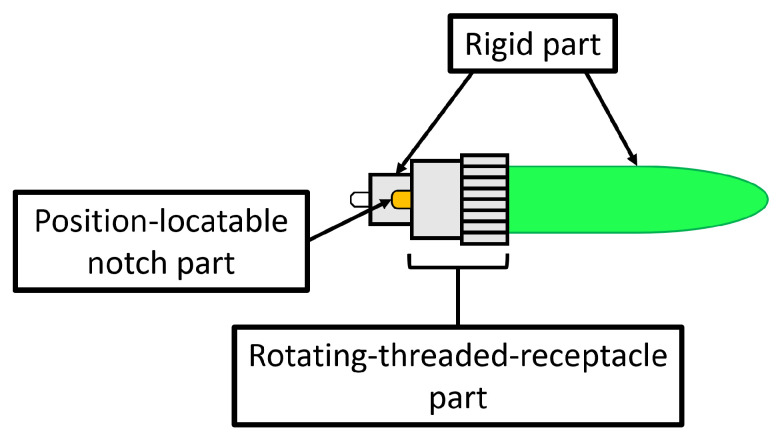
FC connector.

Two kinds of tasks were performed: the unplugging task, i.e., to unplug the FC connector to avoid losing it, and the plugging in task, i.e., to plug in the FC connector. The connector of the optical fibre was made of three parts (Figure 5): the first one was integrated with the cable and then rigid; the second one was the rotating threaded receptacle part useful for screwing/unscrewing the connector, with the specular part blocking them during use; the last one was a position-locatable notch to align male and female connectors.

The normal use of this connector involves the screwing/unscrewing of the rotating part and then the grasping of the rigid part to remove the connector from the female part. An interferometer was connected to the backside of the fibre optic coupler to understand if the unplugging or the plugging in was correct, reading signal interruption for the first task and signal recovery for the second one. Information about the signal from the interferometer was read by employing a PC (left side of Figure 4a).

**Figure 6:**
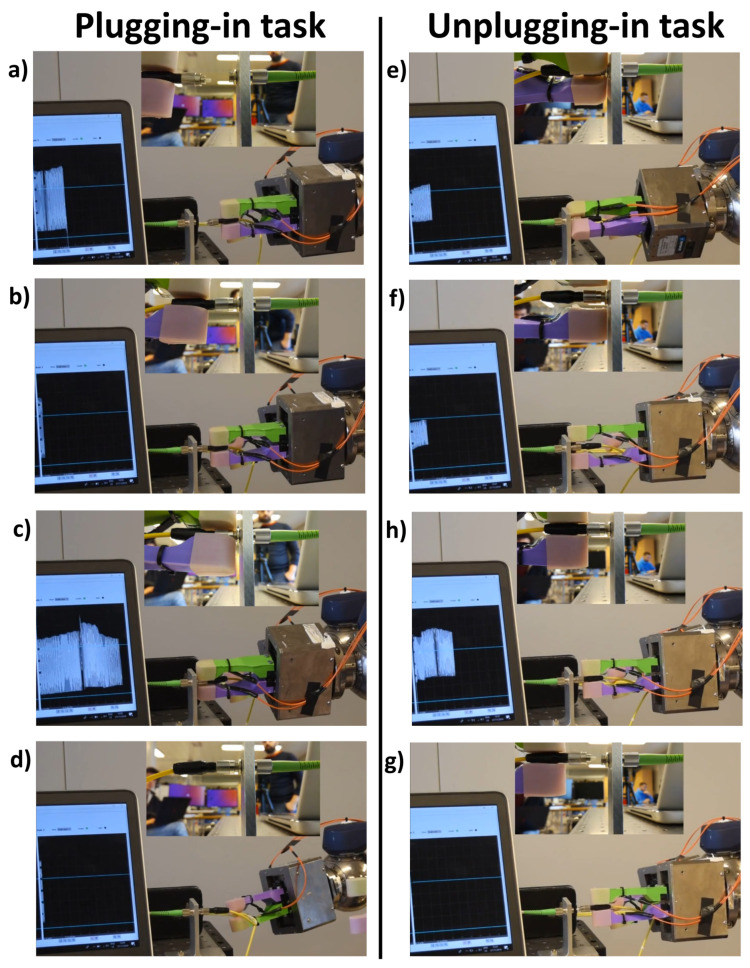
Different phases of the two tasks. (**a**) First step: the two grippers grasp the cable and the FC connector; (**b**) Second step: the left arm moves to plug-in the cable in the fiber optic coupler; (**c**) Third step: the gripper on the left arm releases the rigid part to grasp the rotating-threaded-receptacle part and to screw the connector; (**d**) Fourth step: the gripper on the left arm releases the rotating-threaded-receptacle part. (**e**) First step: the two grippers grasp the cable of the FC connector; (**f**) the gripper on the left arm releases the rotating-threaded-receptacle part; (**g**) the gripper on the left arm grasps the connector on the rigid part; (**h**) the right arm moves to unplug-in the cable.

For the plugging in task, the following steps need to be performed:

- The gripper on the right arm grasps the cable while the gripper on the left side grasps the FC connector by the rigid part (Figure 6a);
- The left arm moves to plug in the cable in the fibre optic coupler, aligning the position-locatable notch and the recess (Figure 6b);
- The gripper on the left arm releases the rigid part to grasp the rotating threaded receptacle part and to screw the connector (Figure 6c);
- The gripper on the left arm releases the rotating threaded receptacle part (Figure 6d).

For the unplugging task, the following steps need to performed:

- The gripper on the right arm grasps the cable while the gripper on the left side grasps the FC connector on the rotating threaded receptacle part to unscrew it (Figure 6e);
- The gripper on the left arm releases the rotating threaded receptacle part (Figure 6f);
- The gripper on the left arm grasps the connector by the rigid part (Figure 6g);
- The right arm moves to unplug the cable (Figure 6h).

For these tasks, 15 naive users (12 males and 3 females, 26 ± 5 years) were selected to perform the two tasks three times. The tasks were carried out using CERNbot and CERNTAURO with and without the two novel modules: the touch-and-slippage (TAS) and the force-and-slippage (FAS) modules. The naive user was supported by an expert user to explain the basic functionality of the robots in the two operative modalities before the first attempt and to provide minimal support during the entire test. An Arduino board for each gripper was used to read the voltage values from the conditioning circuit and to calculate touch and slippage for both fingers. The force applied on the sensor surface caused a mechanical deformation, resulting in resistance variations. Such variations were converted, using a voltage divider, into voltage values ranging from 0 to 5 V [38]. A relationship between the output voltage value from the FSR and the force value was established with statistical characterization [39]. The relation between voltage and force was modelled as reported in [40]. Force, touch and slippage information was delivered to the two developed modules implemented in the CERNTAURO framework to obtain the necessary torques for activating the gripper motion.

## 3. Results and Discussion

**Figure 7:**
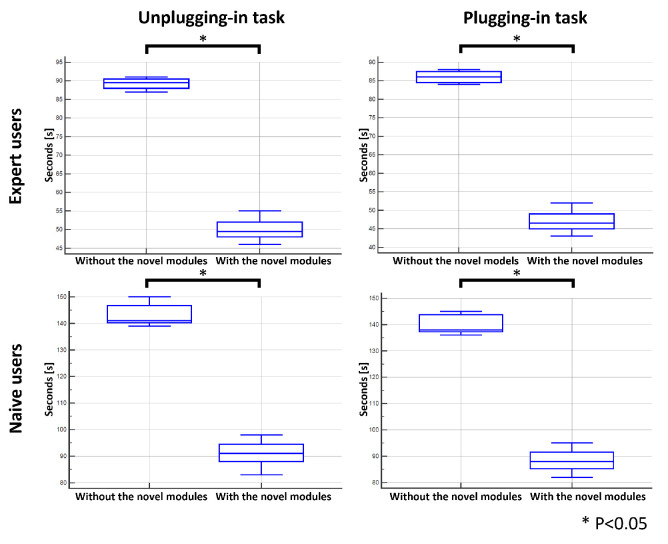
Task execution time (in seconds) for expert and naive operators. Statistical significance when the novel modules were used is indicated with * *p* < 0.05 (Mann–Whitney test).

The users were asked to accomplish the two tasks (to unplug and plug in the cable) three times. Moreover, to provide a baseline for the time comparison, the tasks were executed in both modalities by two expert users; their execution time was considered a lower bound of the execution of the tasks. The difference in the execution time was statistically significant (Mann–Whitney test, *p* < 0.05) for both expert and naive users when the two tasks were performed with the combination of the two novel modules, i.e., TAS and FAS, and the ORM (Figure 7).

**Figure 8:**
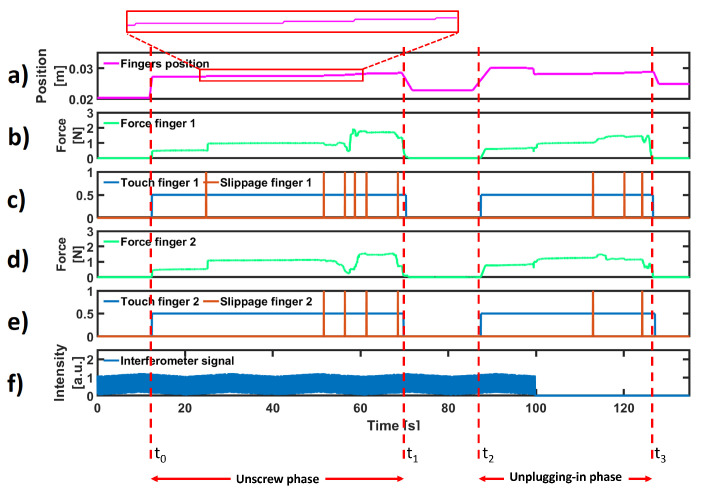
Experimental results for a single unplugging task. (**a**) Position of each finger. (**b**,**d**) Normal forces acquired by the FSR sensors on the fingers. (**c**,**e**) Detected touch and slippage events. (**f**) Interferometer signal.

For the sake of brevity, force, touch, slippage, position and interferometer information is shown in Figure 8 for a single unplugging task. This task was performed in two parts: in the first one, the FC connector was unscrewed (from time $t_{0}$ to $t_{1}$), and in the second one, the fibre was removed from the fibre optic coupler of the test bench (from time $t_{2}$ to $t_{4}$).

Subplot (a) depicts the position of each finger. As described in Section 2.5, the gripper was symmetric, and the displacement of only one finger is reported. The gripper was positioned close to the test bench with an opening similar to the FC connector diameter (∼10 mm) to reduce the time of operation. For this reason, the position of each finger did not start from zero. Subplots (b) and (d) show the forces applied by the fingers. In subplots (c) and (e), the binary signals related to touch and slippage events detected by each finger [25] are reported. In the last subplot, the signal acquired by the interferometer was used to ensure that connector disconnection/connection had been established (in this case, at time $t_{3}$, the loss of interferometer signal to demonstrate the success of the task). In the first phase, the gripper grasped the threaded receptacle, rotated to unscrew it and opened the fingers. In the second phase, it repositioned itself to grasp the rigid part of the connector to remove it from the fibre optic coupler of the test bench. In the first phase, one slippage event was detected, while in the second phase, many of them were detected and contrasted. This is normal behaviour, because the FC connector and the fibre are made of different materials and have different friction coefficients. Hence, the vibrations induced by slippage are different for each phase [41].

To measure the effectiveness (i.e., the ability to obtain the desired results in an ideal context) of using the novel modules, the interferometer signal was taken as a reference (f). When the FC connector was connected to the coupler, i.e., when a good signal transmission through the optical fibre was guaranteed, the amplitude of the interferometer signal had a maximum amplitude between 1.1 and 1.2 values. Therefore, if at the end of the plugging in operation, the connector was between the fingers of the gripper and the signal was less than 1.1, the task was still considered unsuccessful. Again, for the unplugging operation, the task was considered a success when the interferometer signal was equal to 0 and the connector was still between the fingers of the gripper.

**Figure 9:**
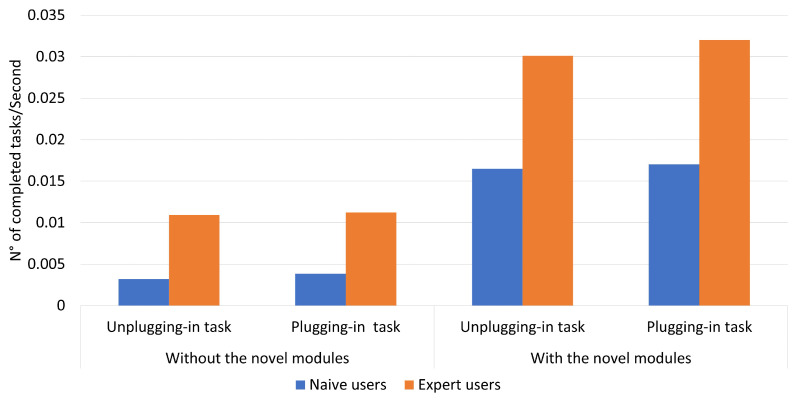
Time-based efficiency of the two tasks performed without and with the two novel modules.

**Figure 10:**
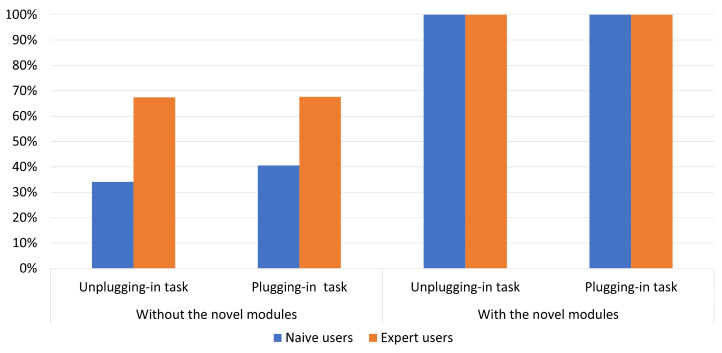
Overall relative efficiency of the two tasks performed without and with the two novel modules.

According to ISO-9241, efficiency represents the resources spent by the users to ensure accurate and complete achievement of the goals. Efficiency is measured in terms of the time employed by the participant to complete a task and can be defined in two ways: time-based efficiency (the measurement of the time spent by the user to complete the task; Figure 9) and the overall relative efficiency of the task (users who completed the task concerning the total time taken by all users; Figure 10) [42].

In particular, the time-based efficiency for expert users was about 0.03 with the combination of the two novel modules as compared with the value obtained in the situation without the new modules, which was half. A similar situation was found for naive users, with values of about 0.01 with the novel modules and less than half without the new modules.

The overall relative efficiency was equal to 100% for both expert and naive users when the two modules were used. Without the novel modules, the percentages were less the 70% for expert users and less the 40% for naive users.

The combined use of the two novel modules, i.e., TAS and FAS, led to a success rate (i.e., No. of completed tasks/No. of tasks) of 100%.

## 4. Comparison with Other Approaches

The analysed works propose different strategies to control a gripper by managing grasping force and slippage.

In [10], the force reference is obtained with the initial grasp, but without lifting the object. Hence, object re-grasping is necessary for the phase immediately following lifting. Instead, the proposed approach detects the needed force to manage the grasped object in an offline phase to avoid a successive re-grasping phase. Moreover, when a slip is detected, a corrective action is performed to make the grasp action more robust. Differently from [11], where friction information is needed to solve an equation for determining the right torque to avoid slipping during grasping, the proposed approach only needs the information of the force reference previously calculated. During the task, only the forces read by the FSR sensors are employed.

Environments such as those where CERNBot is used cannot be equipped with systems to digitally reconstruct the entire workspace (such as optoelectronic systems with markers). Furthermore, human intervention needs to be limited or even forbidden, so the knowledge of the actual positions of each object within the work environment is difficult. This makes the methods described in the Introduction section unsuitable for our purposes.

On the other hand, the knowledge of some information about the operation to perform, such as the connectors to manipulate, is possible. Then, since such operations are planned in advance, the determination of the force references, as well as the recognition of new objects, can be made in the laboratory using bench tests.

## 5. Conclusions

Safety in the work environment is the main advantage of utilizing robotics [43]. Employees who work in hazardous environments can delegate to a robot dangerous tasks that are not possible or safe for humans. The CERNTAURO framework was developed at CERN to help users to perform robotic tasks comfortably, increasing the success rate and safety, and decreasing the intervention time. Teleoperation is currently the only solution to intervene for maintenance in extreme environments. Nevertheless, there are some types of tasks that require particular attention from users. In particular, grasping an optical fibre, and plugging in and unplugging its connector were open challenges.

Until today, the only solution for plugging in or unplugging connectors within hazardous environments was the employment of operators. This involved exposure to conditions dangerous to their health. Tests carried out within the laboratory showed a high failure rate if a robot was used without the solutions described in this work, thus still requiring the intervention of operators.

In this work, two novel modules were added to the CERNTAURO framework (i) to detect touching and slipping between the sensor positioned on the gripper finger and the object surface and to use this information (ii) to regulate the force and to avoid slips during the whole task.

The time-based efficiency when using the combination of the two novel modules was found to be twice as much as that achieved in the same task performed without them. The overall relative efficiency achieved using the two novel modules was 100% for both expert and naive users, while the percentage was less than 70% for expert users and less than 40% for naive users without the new modules. The combination of (i) the two novel modules, (ii) the sensorised fingers mounted on each gripper of the CERNBot platform and (iii) the ORM showed 100% success for both expert and naive users, as evidenced by both effectiveness and efficiency. In addition, users reported a decrease in anxiety due to the task to be performed, as the automatic management of force and slippage allowed them to only focus on plugging in or unplugging the connector.

Future works should test these novel modules during interventions in hazardous environments, such as the LHC [3], the collimator in the North Experimental area [44], the Super Proton Synchrotron accelerator [45] or the Antimatter Production facility [46].

## Figures and Tables

**Table 1:** Force reference to plug in and unplug in the reported connectors.

Connector Type	Manipulation Force (N)
FC	2.3 ± 0.13
Fisher ST-ST	4.14 ± 0.34
LEMO FIG	3.67 ± 0.21
LEMO FZG	3.89 ± 0.16

## Data Availability

Not applicable.
